# Editors’ Book Table

**Published:** 1874-09

**Authors:** 


### Editors' Book Table.

NOTE. — All works reviewed in the columns of the CHICAGO MEDICAL JOURNAL may be found in the extensive stock of W. B. KEEN, COOKE & CO., whose catalogue of Medical Books will be sent to any address upon request.

*A Practical Treatise on the Surgical Diseases of the Genito-Urinary Organs, including Syphilis.* Designed as a Manual for Students and Practitioners. With engravings and cases. By W. H. VAN BUREN, A.M., M.D., Professor of the Principles of Surgery, etc., in Bellevue Hospital Medical College, etc., etc., and E. L. KEYES, A.M., M.D., Professor of Dermatology in Bellevue Hospital Medical College, etc., etc., etc. New York: D. Appleton & Co., 549 and 551 Broadway. 1874.

It is with no small degree of professional and national pride, that we receive this work of the distinguished Professor of Bellevue as another illustration of the rapid advances now being made in medical literature on this side of the ocean—advances which are rapidly diminishing our dependence upon foreign sources of instruction in the more purely scientific departments of medicine.

The work is divided into two sections, the first of which—four hundred and seventy-two pages—is devoted to the consideration of diseases of the genito-urinary organs, including those of the penis, urethra, prostate gland, bladder, ureters, kidneys, testicles, scrotum, spermatic cord and vesicular seminales, with the operative procedures necessary for their relief, and full descriptions of the instruments required. The second section of two hundred pages comprises the various manifestations of syphilis and chancroid. In the first section of the work, the chapter on "urethral and sexual hygiene" deserves to be written in letters of gold, containing, in the short space of two pages, lessons in moral, mental and somatic hygiene, which, if faithfully applied, would soon rid the world of a large class of diseases.

The treatise on prostatic disease is unusually full, and constitutes the most valuable contribution to this department of pathology in the English language. The description of that rare form of disease, tubercular kidney, has evidently been written from practical observation of the disease, and to one familiar with its morbid anatomy is singularly accurate.

On page 169, the "summary of treatment of stricture" is more comprehensive than many large volumes.

The author declines, very wisely, the discussion of the vexed question of the unity or duality of the syphilitic poison, upon which so much bad logic has been expended by medical writers. He, however, practically takes position with the unicists, inasmuch as he describes most carefully, syphilis in its various forms and modes of manifestation, and equally carefully another disease which is not syphilis but "chancroid," lacking some of the essential characteristics of syphilis. This question of unity or duality of syphilis has always appeared to us as a parallel to that concerning "true" and "spurious" vaccine. That is to say, there is a disease whose specificity depends upon the presence of certain characteristic symptoms and appearances. These absent, the essentials of the disease no longer existing, the disease itself is not, but instead, we have something essentially, specifically distinct, which may be designated as may seem best, but should be correlated with the first only antithetically.

On page 555, we are glad to see that Dr. Van Buren speaks unequivocally upon this head. In speaking of that most illogical, irrational mode of treatment, syphilization, he says, "its premises are scientifically inexact, for *chancroid* is not syphilis any more than is nettlerash the itch."

The remarks in the latter part of the volume upon syphilis of the nervous system, shed light upon much that was obscure in this peculiar department of pathology. Taken altogether, the work must take rank amongst the classics of professional literature.

The mechanical portion of the work cannot be commended more highly than by saying that it is issued by the great house of D. Appleton & Co. H.

*Observations on the Pathology and Treatment of Cholera,—The Result of Forty Years' Experience.* By JOHN MURRAY, M.D., Inspector-General of Hospitals, late of Bengal. New York: G. P. Putnam's Sons. 1874.

A record of the accumulated experience of forty years in the observation and treatment of any disease could scarcely fail to contain much valuable information, and hence it is to be regretted that the author has condensed his subject so severely that much valuable matter must necessarily have escaped.

In fifty duodecimo pages it is scarcely possible to give more than the barest epitome of the observations which the most careless observer must have made during forty years in a locality so prolific in examples of any disease as India of cholera. It is rare that the reviewer is called upon to complain of the brevity of an author. In this case, one of the advantages may be, that every one will read it, as every one should, and every one who reads will have but one comment, like that upon the old lady's pudding, "very good! what there is of it." H.

*A Universal Formulary.* Containing the Methods of Preparing and Administering Officinal and other Remedies; the whole adapted to Physicians and Pharmacists. By R. EGLESFELD GRIFFITH, M.D. Third edition, carefully revised and much enlarged by JOHN MAISCH, Phar. D., Professor of Materia Medica and Botany in the Philadelphia College of Pharmacy. With illustrations. Philadelphia: Henry C. Lea. 1874.

Griffith's Medical Formulary is so well known to the profession as to require but a passing notice. A work more useful in its especial department cannot be found, the formulæ being derived from the highest authorities, and duly accredited. There are occasional defects to be found, as nothing is perfect, but the formulæ on page 557, credited to Magendie, we think would scarcely be deemed safe by those accustomed to the use of strychnia, *i. e.*, one-twelfth to one-sixth of a grain morning and evening. Nor would the two following, which permit the use of this drug in doses of one-tenth to one-sixth of a grain, be altogether free from danger. Whereas the formulæ of Giubourt and Brera, for one-sixteenth and one-twentieth (respectively) of nitrate of silver, can scarcely be considered sufficiently active to possess any efficiency. The formula from Bouchardat, (atropia 15 grs., alcohol 10 fl. drachms, dose one to three drops,) is dangerous, one to three drops of the tincture containing one-fortieth to one-thirteenth (nearly) of a grain of the alkaloid; the minimum of these should be the maximum. These are incidental defects, and do not detract from the general merits of the book, which, in the main, is beyond criticism. The editorial excellence of the work is guaranteed by the name of Jno. M. Maisch, and its mechanical and typographical perfection by the imprimatur of Henry C. Lea. H.

BOOKS RECEIVED.

*Nomenclature of Disease.* Prepared for the use of the Medical Officers of the United States Marine Hospital Service, by the Supervising Surgeon, JNO. M. WOODWORTH, M.D.

*Essays on Conservative Medicine and Kindred Topics.* By AUSTIN FLINT, M.D., Prof. of the Principles and Practice of Medicine and of Clinical Medicine in the Bellevue Hospital Medical College, N. Y.

PAMPHLETS RECEIVED.

*A New Method of Treating Malignant Tumors by Electrolyzing the Base.* By GEORGE BEARD, M.D. New York. 1874.

*Atmospheric Electricity and Ozone: Their Relations to Health and Disease.* By GEORGE M. BEARD, M.D.

*Catalogue and Announcement of the Medical Department of the University of Pennsylvania for the 109th Session,* 1874-75.

*Eighth Annual Announcement of the Chicago College of Pharmacy.*

*On the Value of High Powers in the Diagnosis of Blood Stains.* By JOSEPH G. RICHARDSON, M.D., Lecturer on Pathological Anatomy in the University of Pennsylvania, and Microscopist to the Pennsylvania Hospital.

*The Vanderbilt University Department of Medicine and Surgery.*

*Transactions of the Eighth Annual Meeting of the Medical Association of the State of Missouri, held at Sedalia, April* 21, 1874.

*Report on the Progress of Surgery.* By J. W. TRADER, M.D., Sedalia, Mo.

*Direct Local Medication in the Treatment of Chronic-Catarrhal Inflammation of the Nasal and Pharingo-Nasal Cavities.* By THOMAS F. RUMBOLD, M.D., St. Louis.

JOURNALS RECEIVED.

L'Anatomie et de la Physiologie, Journal de M. Charles Robin, Paris—Juilliet et Aout.
The Atlanta Medical and Surgical Journal—August.
" American Medical Weekly, Louisville—Nos. 1, 2, and 3.
" American Journal of Syphilography and Dermatology—July.
" Archives of Ophthalmology and Otology—Vol. IV, No. 1.
" Braithwaite's Retrospect—July, 1874.
" Boston Medical and Surgical Journal—August 1.
" Buffalo Medical and Surgical Journal—June.
" Canada Lancet—August 1.
" Cincinnati Lancet and Observer—August.
" Clinic—July 18, 25.
" Canada Medical and Surgical Journal—July.
" Chicago Journal of Nervous and Mental Disease—July, 1874.
" Druggists' Circular—August 1.
" Detroit Review of Medicine—August.
" Dental Cosmos—August.
" Kansas City Medical Journal—July.

The London Lancet—July.
" Medical and Surgical Reporter—July 18, 25, August 1, 8.
" Medical Record, New York—July 15, August 1.
" Medical Science, Half-Yearly Compendium of, Philadelphia—July, 1874.
" Medical Times, Philadelphia—July 11, 18, 25, Aug. 1, 8.
" Medical Examiner, July 15, August 7.
" Medical Press and Circular, London—July.
" Medical and Surgical Review, (Australasian)—May.
" Medical News and Library—August, 1874.
" Medical News and Library, Supplement.
" Missouri Clinical Record—August.
" New Remedies. A Quarterly Retrospect of Therapeutics, Pharmacy, etc.—July.
" Nashville Journal of Medicine and Surgery—July and August.
" New York Medical Journal—August, 1874.
" Psychological and Medico-Legal Journal, New York—July.
" Pacific Medical and Surgical Journal—July.
" Peninsular Journal of Medicine—August.
" Practitioner, London—July.
" Pharmacist—August.
" Richmond and Louisville Medical and Surgical Journal—July.
" Southern Medical Record—July.
" St. Louis Medical and Surgical Journal—August.
" Technologist—July.
" Virginia Medical Monthly—August, 1874.

